# Sequence-Based Prediction of RNA-Binding Proteins Using Random Forest with Minimum Redundancy Maximum Relevance Feature Selection

**DOI:** 10.1155/2015/425810

**Published:** 2015-10-12

**Authors:** Xin Ma, Jing Guo, Xiao Sun

**Affiliations:** ^1^Golden Audit College, Nanjing Audit University, Nanjing 210029, China; ^2^State Key Laboratory of Bioelectronics, Southeast University, Nanjing 210096, China

## Abstract

The prediction of RNA-binding proteins is one of the most challenging problems in computation biology. Although some studies have investigated this problem, the accuracy of prediction is still not sufficient. In this study, a highly accurate method was developed to predict RNA-binding proteins from amino acid sequences using random forests with the minimum redundancy maximum relevance (mRMR) method, followed by incremental feature selection (IFS). We incorporated features of conjoint triad features and three novel features: binding propensity (BP), nonbinding propensity (NBP), and evolutionary information combined with physicochemical properties (EIPP). The results showed that these novel features have important roles in improving the performance of the predictor. Using the mRMR-IFS method, our predictor achieved the best performance (86.62% accuracy and 0.737 Matthews correlation coefficient). High prediction accuracy and successful prediction performance suggested that our method can be a useful approach to identify RNA-binding proteins from sequence information.

## 1. Introduction

RNA-binding proteins are important functional proteins that are pivotal to a cell's function, such as in gene expression, posttranscriptional regulation, protein synthesis, and replication and assembly of many viruses [1–4]. How to discriminate RNA-binding proteins from other proteins is important to understand the mechanisms of these functions. Therefore, the reliable identification of RNA-binding proteins is an important research topic in the field of proteomics and will play a vital role in proteome functional annotation, in the discovery of potential therapeutics for genetic diseases and in reliable diagnostics. Several experimental techniques, such as X-ray crystallography, nuclear magnetic resonance, and filter binding assays have been used to identify RNA-binding proteins. However, using experimental methods to identify RNA-binding proteins is costly and time consuming. It is desirable to develop computational methods to recognize RNA-binding proteins.

Previous studies have investigated the mechanisms by which proteins bind to DNA; however, research on RNA-binding proteins lags behind. Methods to identify RNA-binding proteins could be divided into two categories: recognition from protein structure and prediction from amino acid sequences. The structure-based prediction approach usually produces a better performance; however, obtaining the protein structure is still costly and time consuming. Considering the theory that a protein's amino acid sequence contains all the necessary information to predict its function [5], we hypothesized that it would be an effective approach to predict RNA-binding proteins directly from amino acid sequences. Therefore, machine learning algorithms have been used to build classification systems to discriminate RNA-binding proteins from nonbinding ones.

Support vector machine (SVM) [6] is an effective machine learning algorithm that is the most widely used for prediction of RNA-binding proteins [7–11]. Cai and Lin first built a prediction model by using SVM and incorporated a comprehensive set of input features based on the amino acid composition and a limited range of correlations with hydrophobicity and solvent accessible surface area [7]. Shao et al. proposed an SVM-based predictor using a conjoint triad feature, which extracts information directly from the amino acids sequences of proteins [10]. Kumar et al. developed a prediction model named RNApred (http://www.imtech.res.in/raghava/rnapred/), which also uses an SVM and uses a position-specific scoring matrix (PSSM) profile and sequence descriptors as inputs [11].

To obtain a good predictive model, two major problems should be considered. One is feature extraction and selection and the other is the selection of the classification algorithm. To solve the first problem, we proposed a novel feature called evolutionary information combined with physicochemical properties (EIPP). The results show that EIPP has a more powerful ability to distinguish RNA-binding proteins from nonbinding ones than PSSM, which dramatically improved the prediction of RNA-binding proteins compared with a previous work [11]. In our study, we used the minimum redundancy maximum relevance (mRMR) method combined with incremental feature selection (IFS) to select the optimal features, which not only reduced the dimension of the features but also improved the performance of the predictor. To solve the second problem, we choose the random forest (RF) algorithm [12] instead of the SVM algorithm because the SVM algorithm is time consuming when searching for appropriate optimal parameters, and the kernel function for the predictor and RF algorithm is an ensemble classifier with fast performance that has been applied successfully in many fields. Therefore, in this study, the mRMR-IFS feature selection approach and the RF algorithm are combined to construct the prediction model. Our results showed that the prediction model achieved 86.62% accuracy, 78.34% sensitivity, and 94.91% specificity, with a Matthews correlation coefficient of 0.737, indicating that it outperformed previous methods in predicting RNA-binding proteins.

## 2. Materials and Methods

### 2.1. Dataset

RNA-binding proteins and nonbinding proteins were obtained from release “2014_06” of the UniProtKB database (http://www.uniprot.org/) [13]. By searching with the keyword “RNA binding,” we extracted 47,768 RNA-binding proteins from UniProtKB. We followed the procedure by Yu et al. [9] to obtain 545,536 nonbinding proteins. To ensure the reliability of data, we only selected manually annotated and reviewed proteins.

As indicated by previous studies [8–10], the data used in this study were selected strictly according to the following criteria. (1) Protein sequences with more than 6000 amino acids were removed because they might be protein complexes. Protein sequences less than 50 amino acids were also removed because they might be protein fragments. (2) Proteins including irregular amino acid characters such as “x” and “z” were filtered out. (3) To reduce redundancy and homology bias, the BLAST package was used in this research. The BLAST package was downloaded from NCBI utilized to remove those sequences that have 40% sequence identity to any other sequences in the dataset. To create the nonredundant dataset, the longest amino acid sequences were selected in each cluster. Finally, we obtained 2,848 RNA-binding proteins as positive instances and 83,516 nonbinding proteins as negative instances. To achieve a balance between positive instances and negative instances, we randomly selected the same number of negative instances as the number of positive instances. Therefore, the main dataset (MDset) used in this study comprised 2,848 RNA-binding proteins and 2,848 nonbinding proteins.

To evaluate the performance of our method in comparison with previously well-known studies, we used an independent test dataset (Testset). The Testset comprised 144 RNA-binding proteins and 144 nonbinding proteins obtained from MDset that had not been used in previous studies [11, 14]. The remaining proteins in MDset were designated as the training dataset (TRset). Therefore, TRset contained 2,704 RNA-binding proteins and 2,704 nonbinding proteins.

### 2.2. Protein Features

#### 2.2.1. Binding Propensity and Nonbinding Propensity (BP and NBP)

Prediction of RNA-binding residues was used to identify the RNA-binding proteins from nonbinding ones. We had already developed an RNA-binding residues prediction model, PRBR [15] (http://www.cbi.seu.edu.cn/PRBR/). Each amino acid could be identified by submitting the protein to the PRBR webserver. Consequently, the binding propensity measures and nonbinding propensity measures were adopted in this study, which were made based on the prediction results of RNA-binding residues and nonbinding residues, respectively.

RNA-binding proteins have many more binding residues than nonbinding proteins and RNA-binding residues tend to gather together spatially; therefore, two binding propensity measures were defined as follows:

(1) $$\begin{array}{c} BP\left(\;1\;\right)=\frac{\sum_{i=1}^{n}RI\left(i\right)}{10N}, \end{array}$$

where *N* and *n* are the number of amino acids in this protein and the number of RNA-binding residues, respectively. RI(*i*) is the reliability index of the prediction result of RNA-binding residue *i* obtained from PRBR. The reliability index is a positive integer ranging from 0 to 10. Consider

(2) $$\begin{array}{c} BP\left(\;2\;\right)=\frac{\sum_{i=1}^{N-1}2^{-i+1}\sum_{k=1}^{n\left(i\right)}\bar{RI}\left(k\right)}{10\left(N-1\right)}, \end{array}$$

where *N* and *n*(*i*) are the number of amino acids in this protein and the number of two RNA-binding residues at a distance *i*, respectively. $\bar{RI}\left(i\right)$ is the average value of the reliability index for RNA-binding residue *k* and binding residue *k* + *i*.

We used predicted RNA-binding residues; therefore, the reliability index is applied in those two formulas. The BP(1) and BP(2) represent the information of the frequency and correlation of RNA-binding residues in the query protein, respectively. Furthermore, BP(2) formula represents the relevance of the two RNA-binding residues combined with different distances from 1 to *N* − 1 and takes into account the fact that the correlation value between two residues is smaller when the distance *k* is larger which proves the rationality of the definition.

We also defined two nonbinding propensities for nonbinding proteins. The definitions of NBP(1) and NBP(2) are similar to the definitions of BP(1) and BP(2). Consider

(3) $$\begin{array}{c} NBP\left(\;1\;\right)=\frac{\sum_{i=1}^{n}RI\left(i\right)}{10N}, \end{array}$$

where *N* and *n* are the number of amino acids and the number of nonbinding residues in this protein, respectively. RI(*i*) is the reliability index of the prediction result of nonbinding residue *i* obtained from PRBR. Consider

(4) $$\begin{array}{c} NBP\left(\;2\;\right)=\frac{\sum_{i=1}^{N-1}2^{-i+1}\sum_{k=1}^{n\left(i\right)}\bar{RI}\left(k\right)}{10\left(N-1\right)}, \end{array}$$

where *N* is the number of amino acids in this protein, *n*(*i*) is the number of two nonbinding residues at a distance *i*, and $\bar{RI}\left(k\right)$ is the average value of the reliability index for nonbinding residue *k* and nonbinding residue *k* + *i*.

NBP(1) and NBP(2) describe the information of the appearance and correlation of nonbinding residues in the query protein, respectively, which are similar to BP(1) and BP(2). We also used the reliability index because the prediction result of nonbinding residues is applied in those formulas.

#### 2.2.2. Evolutionary Information Combined with Physicochemical Properties (EIPP)

Evolutionary information in the form of a position-specific scoring matrix (PSSM) has been used successfully to represent proteins in many applications, such as prediction of DNA-binding residues [16–21] and RNA-binding residues [15, 22, 23]. Here, PSSM profiles were generated using the PSI-BLAST program [24] to search the nonredundant (NR) database through three iterations, with 0.001 as the *e*-value cutoff for multiple sequence alignment. The PSSM scoring matrix has 20*∗L* elements, where *L* is the length of protein. However, different proteins may have different numbers of amino acids. Therefore, the PSSM could not be used directly as feature in the prediction work because all the machine learning methods require the input feature to have a fixed length. Therefore, we generated a PSSM-400, which has a vector of dimension of 400 from the PSSM. PSSM-400 is composition of occurrences of each type of amino acid corresponding to each type of amino acids in sequences. We pooled all rows that belonged to the same amino acid in this PSSM to form a new matrix. We then converted each new matrix to a vector and added all the normalized values in each column for the new matrixes. Therefore, we produced a 20-dimensional vector for each new matrix to generate PSSM-400.

The physicochemical property feature has been used effectively in many fields, such as the identification of DNA∖RNA-binding proteins [7, 9, 14, 25, 26] and the identification and prediction of protein-protein interactions [27]. Thus, an EIPP was generated by merging 20 amino acid columns of the PSSM-400 into a single column containing the information for a certain physicochemical property. Six physicochemical properties that we used successfully in previous works [15] were considered for combining with PSSM-400 to generate the EIPP: the pKa values of the amino group, the pKa values of the carboxyl group [28], the molecular mass [6], the lowest free energy [29], the Balaban index [30], and the Wiener index [31]. The entry *e*_*ak*_ of *k*th type of amino acid in a protein sequence for a certain physicochemical property *a* in EIPP was calculated with

(5) $$\begin{array}{c} e_{ak}=\overset{20}{\underset{i=1}{\sum }}\sqrt{d_{a}\left(i\right)}f_{k}\left(\;i\;\right), \end{array}$$

where *a* is the index of a certain physicochemical property, *k* is the index of the type of amino acids in the query protein sequence, *i* is the index of the type of naïve amino acids, *f*_*k*_(*i*) is the normalized value of the *i*th type of naïve amino acid for the *k*th type of amino acid in the protein sequence of the PSSM-400, and *d*_*a*_(*i*) is the normalized physicochemical property values of *a* for the *i*th type amino acids. Therefore, the vector size of EIPP feature is 6 × 20.

#### 2.2.3. Conjoint Triad (CT)

Electrostatic and hydrophobic interactions influence protein-nucleic acid interactions and may be reflected by the dipoles and volumes of the side chains of amino acids, respectively. Based on the dipoles and volumes of the side chains, the 20 kinds of amino acids could be clustered into seven classes [32]. Considering that disulfide bonds have no special effect on protein-nucleic acid interactions, the unique amino acid cysteine in the seventh class was put back to the third class in this study. Therefore, the 20 kinds of amino acids were clustered into six classes as follows. Class a: Ala, Gly, and Val; Class b: Ile, Leu, Phe, and Pro; Class c: Tyr, Met, Thr, Ser, and Cys; Class d: His, Asn, Gln, and Tpr; Class e: Arg and Lys; and Class f: Asp and Glu. According to the similar feature construction method used in [32], a protein is described by the conjoint triads feature with 6 × 6 × 6 = 216 dimensions, where each component of the feature vector has the value of the frequency of the corresponding triad.

As mentioned above, for each query protein, the vector size of a feature is 4 + 120 + 216 = 340.

### 2.3. Algorithms to Classify and Measure a Classifier's Performance

The random forest (RF) algorithm [12] is a classification algorithm that uses an ensemble of tree-structured classifiers, which has been used successfully in many applications for data classification and achieves high performance. The random forest R package [33] was used to implement the RF algorithm.

To evaluate the performance of the classifier, a 5-fold cross-validation procedure for the training dataset was used in this research. During the procedure, we randomly divided the data instances into five parts. Four of these parts were input into the RF to establish a model for classification, and every instance of the remaining part was predicted by the model. Ultimately, the prediction performance of the classifier was evaluated by the remaining part.

To evaluate the performance of the RNA-binding proteins predictor, the accuracy, sensitivity, specificity, and Matthews correlation coefficient (MCC) were calculated as follows:

(6) Accuracy=TP+TNTP+TN+FP+FN,Sensitivity=TPTP+FN,Specificity=TNTN+FP,MCC=TP×TN−FP×FNTP+FPTN+FNTP+FNTN+FP,

where TP, TN, FP, and FN represent the number of true positive, true negative, false positive, and false negative results, respectively.

### 2.4. Minimum Redundancy Maximum Relevance (mRMR) and Incremental Feature Selection (IFS)

Considering the successful application on several classification researches [34–42] by using the minimum redundancy Maximum relevance (mRMR) method combining with incremental feature selection (IFS) method, the mRMR-IFS was used in this research to select the prominent features that distinguish the RNA-binding proteins from nonbinding ones.

The mRMR method was developed by Peng et al. [43]. Here, we used it for feature analysis and selection. It selects candidate features with both the maximum relevance for the target and the minimum redundancy relative to the features already selected. To calculate relevance and redundancy, we used mutual information (MI), which is defined as follows:

(7) $$\begin{array}{c} MI\left(\;x,y\;\right)=\iint p\left(\;x,y\;\right)\log\frac{p\left(x,y\right)}{p\left(x\right)p\left(y\right)}\;dx\;dy. \end{array}$$

In (7), *p*(*x*, *y*) is the joint probabilistic density of random vectors *x* and *y*, and *p*(*x*) and *p*(*y*) are the marginal probabilities.

Let *Ω*, *Ω*_*s*_, and *Ω*_*t*_ denote the whole feature set, the already-selected feature set containing *m* features, and the to-be-selected feature set containing *n* features, respectively.

To obtain the feature *f*_*t*_ in *Ω*_*t*_ with the maximal relevance for the target *c* and the minimal redundancy relative to the features in *Ω*_*s*_, the mRMR function is defined as

(8) ft=maxfj∈Ωt⁡MIfj,c−1m∑fi∈ΩsMIfj,fi,j=1,2,…,n.

In this study, after using the mRMR method, all of the 340 features were ordered as follows:

(9) $$\begin{array}{c} S=\left\{\;f_{1}^{\prime },f_{2}^{\prime },\ldots ,f_{h}^{\prime },\ldots ,f_{340}^{\prime }\;\right\}. \end{array}$$

In (9), the earlier the feature satisfying (8), and the smaller the index *h*, the better the feature.

To select the optimal features, we used incremental feature selection (IFS) [35, 36], which is based on the results of mRMR. We first built 340 feature sets from the ordered feature set *S* (9), with the *i*th feature set being

(10) $$\begin{array}{c} S_{i}=\left\{\;f_{1}^{\prime },f_{2}^{\prime },\ldots ,f_{i}^{\prime }\;\right\},\;\left(i=1,2,\ldots ,340\right). \end{array}$$

We then constructed 340 individual predictors for the 340 feature sets to predict RNA-binding proteins. Each predictor was constructed by the RF algorithm and evaluated by 5-fold cross-validation. The 340 MCC values were calculated from all the predictors and obtained the IFS curve with feature index *i* of *S*_*i*_ as the *x*-axis and the MCC value as the *y*-axis. Finally, the optimal feature set was obtained when the IFS curve reached its peak.

## 3. Results and Discussion

### 3.1. Prediction of RNA-Binding Proteins Using Various Features

We explored the performance of RF-based predictors for predicting RNA-binding proteins by various features. The prediction results of the individual RF-based predictors using 10 cycles of 5-fold cross-validation over the MDset are shown in Table 1. First, three features, including PSSM-400, EIPP, and CT, were used to construct RF predictors to predict RNA-binding proteins. As shown in Table 1, the classifier using EIPP achieved a higher performance than the other predictors using a single feature, with 83.11% accuracy and an MCC of 0.662. Therefore, we proposed that EIPP, which provides the evolutionary information and physicochemical properties information of the protein, could effectively distinguish RNA-binding proteins from nonbinding ones. It was obvious that the EIPP features are more powerful than the commonly used PSSM-400. Therefore, we used EIPP as a significant feature instead of PSSM-400 in this study. Although the vector dimensions of the BP and NBP features are the lowest of all the features, they play an important role in improving the performance of the classifier. When the BP and NBP features were combined with EIPP, the accuracy and MCC increased dramatically to 84.28% and 0.704, respectively. When they were combined with CT, the accuracy and MCC also increased, to 76.61% and 0.568, respectively, which are not as good as the performance obtained by the combination of the EIPP, BP, and NBP. Finally, we found that the combination of EIPP, BP, NBP, and CT achieved the best performance, with the results for accuracy, sensitivity, specificity, and MCC of 85.73%, 77.64%, 94.24%, and 0.729, respectively. Thus, the mRMR-IFS method was used to select an optimal feature set from all features, including EIPP, BP, NBP, and CT in this study.

### 3.2. mRMR Results

We ranked a list of 340 features for MDset dataset using the mRMR method, which was downloaded from http://penglab.janelia.org/proj/mRMR/index.htm. Within this mRMR list, a smaller index value for a feature represents higher importance in the prediction of RNA-binding proteins. The ranked 340-feature list was then used in the IFS procedure for optimal feature selection and analysis.

### 3.3. IFS Results

**Figure 1:**
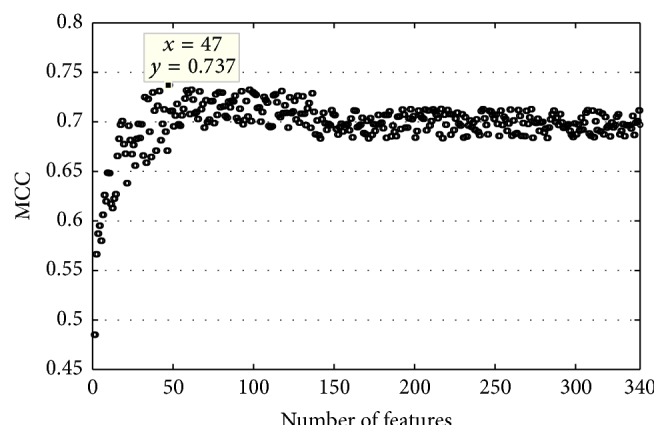
The IFS curve showing MCC values against feature numbers. The maximum MCC value was 0.684 when the top 47 features were selected.

Based on the list of 340 features obtained from the mRMR method, we obtained 340-feature subsets. We then built 340 individual predictors for the 340-subfeature sets to predict RNA-binding proteins, evaluated by 5-fold cross-validation on the MDset dataset. As shown in Figure 1, the IFS curve was plotted by feature indices and MCC values obtained from the corresponding predictor. Using the top 47 features, the maximum MCC value was 0.737. Using these 47 features, the performance of the predictor was better than that of the predictor using all 340 features, with the results for accuracy, sensitivity, specificity, and MCC increasing to 86.62%, 78.34%, 94.91%, and 0.737, respectively. Therefore, these 47 optimal features were considered as the optimal feature set to be used in our final prediction model for predicting RNA-binding protein. The 47 optimal features are shown in Table 2.

### 3.4. Analysis of 47 Features in the Optimal Feature Set

#### 3.4.1. Analysis of the Optimal Feature Set

**Figure 2:**
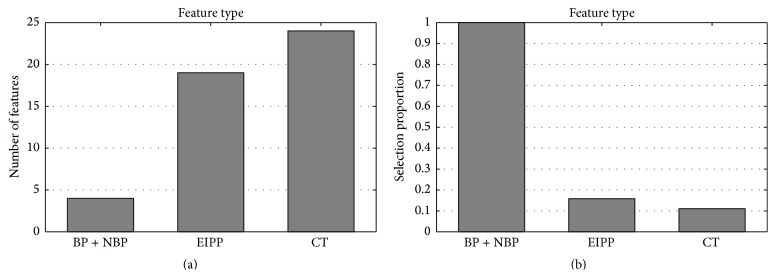
(a) Feature distribution for the 47 optimal features. (b) The selection proportion of each type of feature.

As described in Section 2, there are three types of features in this study, namely, BP/NBP, EIPP, and CT. For the MDset dataset, all of the three types of features with 340 dimensions were reduced to 47 dimensions after mRMR-IFS feature selection process. The number of each type of feature in the optimal feature set is shown in Figure 2(a). The selection proportion of each type of feature for the corresponding type of feature is shown in Figure 2(b).

As shown in Figure 2(a), there were four BP and NBP features, 19 EIPP features and 24 CT features. Although the number of BP and NBP features in the optimal feature set was four, which was the least among the three types of features, the number of BP and NBP features in the original feature set was also four; thus, the selection proportion of BP and NBP features was 100%. This result showed that BP and NBP play an important role in distinguishing RNA-binding proteins from nonbinding ones. The EIPP features and CT features have similar numbers in the optimal feature set. However, the selection proportion of EIPP features (15.83%) is almost one and half times as many as the selection proportion of CT features (11.11%). This result indicated that EIPP also plays a vital role in RNA-binding proteins prediction and that CT contributes the least to the prediction of RNA-binding proteins, which is consistent with the result obtained from Table 1.

#### 3.4.2. Analysis of BP and NBP Features in the Optimal Feature Set

All four BP and NBP features in the original feature dataset were selected to the optimal feature set, which revealed that BP and NBP features contribute mostly to distinguish RNA-binding proteins from nonbinding ones. We also calculated the *p* values of BP and NBP features between the binding proteins and the nonbinding ones to measure the discrimination ability. Each of them was less than 0.00005. These results also proved that BP and NBP could successfully discriminate between DNA-binding proteins and nonbinding proteins.

The superior performance of BP and NBP features represents the reliability of the definition of BP and NBP features. The detailed explanation for the reliability of the definitions of BP and NBP could be as follows. Compared with nonbinding proteins, RNA-binding residues should show a higher tendency to exist in binding proteins and RNA-binding residues should tend to gather together spatially on the surface of an RNA-binding protein. The two BP features revealed the character of RNA-binding proteins at the sequence level and the spatial level, respectively. By contrast, the proportion of nonbinding residues should be much higher for nonbinding proteins in comparison to RNA-binding proteins. This phenomenon represents the reliability of the proposed NBP feature. Therefore, BP and NBP features worked well, as we expected.

#### 3.4.3. Analysis of EIPP Features in the Optimal Feature Set

**Figure 3:**
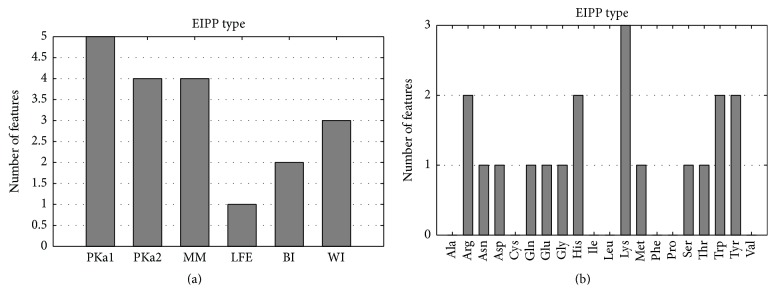
(a) Physicochemical property distribution to construct the 19 EIPP features that were selected in the optimal feature set. (b) The type of amino acids distribution to construct the 19 EIPP features that were selected in the optimal feature set.

We selected 19 EIPP features in the optimal feature set after using the mRMR-IFS method. Considering that EIPP was constructed by the evolutionary information of each type of amino acid in sequences and physicochemical property, we collected the statistics of the number of each type of amino acid and the number of each type of physicochemical property that constituted the 19 EIPP features. Figures 3(a) and 3(b) show the contributions of the number of each type of physicochemical property and the number of each type of amino acid, respectively.

As seen from Figure 3(a), there are five features related to the pKa values of amino group (PKa1), four features related to the pKa values of carboxyl group (PKa2), four features related to the molecular mass (MM), one feature related to the lowest free energy (LFE), two features related to the Balaban index (BI), and three features related to the Wiener index (WI). Compared with all physicochemical properties used in EIPP, PKa1 and PKa2, which determine the ionization state of a residue, are most essential for protein-RNA interaction. The reason is that the ionization state of amino acid side chains affects the interaction with RNA molecules, which have negatively charged phosphate groups. Molecular mass is irreplaceable in protein-RNA interactions because it is related to the volume of space that a residue occupies in the structure. The topological indices of a molecule, such as the Wiener index and the Balaban index, also play an important role in binding activity.  Figure 3(b) shows that lysine, arginine, histidine, tryptophan, and tyrosine most frequently constituted the 19 EIPP features. This is most likely because those types of amino acids are abundant in RNA-binding sites and show the highest binding propensities for RNA-protein interactions. This is consistent with several results obtained from previous studies [44–46]. Lysine, arginine, and histidine show the highest binding tendency because they are positively charged amino acids and can easily interact with the negatively charged phosphate backbone of RNA.

#### 3.4.4. Analysis of CT Features in Optimal Feature Set

**Figure 4:**
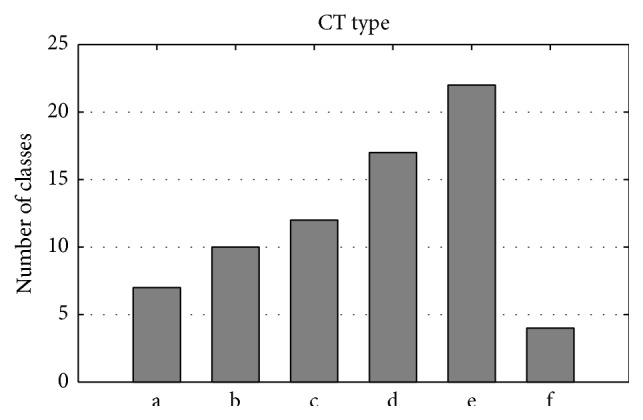
The type of class distribution to construct the 24 CT features that were selected in the optimal feature set.

Twenty-four CT features were selected in the optimal feature set and the number of each type of class, which constituted the 24 CT features, was analyzed and shown in Figure 4. There are 72 classes comprising the 24 CT features. Classes e and d show the highest occurrence numbers, 22 and 17, respectively. The result is rational, because lysine and arginine belong to class e, which showed the easiest interaction ability with RNA. Class c appeared 12 times in the 24 CT features, which ranked third among the six classes, perhaps because class c has five types of amino acids, the most number of types of amino acids among six classes. Class f occurred the least frequently, at four times in 24 CT features. This is because glutamate and aspartate, which constitute class f, are negatively charged amino acids, which would find it harder to interact with the negatively charged phosphate backbone of RNA.

### 3.5. Comparison with Existing Methods on an Independent Dataset

To evaluate the effectiveness of our protocol, we compared the performance of our method with existing methods. Currently, there are two webservers for identifying RNA-binding proteins based on sequence information. One is SVMprot by Han et al. [8] (http://jing.cz3.nus.edu.sg/cgi-bin/svmprot.cgi), which predicts RNA-binding proteins using SVM with encoded representations of tabulated residue properties as features. The other is RNApred by Kumar et al. [11] (http://www.imtech.res.in/raghava/rnapred/), which predicts RNA-binding proteins by SVM with PSSMs. To ensure that the comparison result is fair, a dataset Testset, with 288 proteins, was used as an independent test dataset, which did not include the proteins mentioned in [8, 11]. The mRMR-IFS selection model was reconstructed based on the training dataset TRset and used to predict the putative RNA-binding proteins in the Testset. Our method correctly predicted 117 out of 144 RNA-binding proteins and 104 out of 144 nonbinding proteins. Then we submitted the proteins in Testset to SVMprot. Out of 144 RNA-binding proteins, SVMprot correctly predicted 56 as RNA-binding proteins. Out of 144 nonbinding proteins, it correctly predicted 110 as nonbinding proteins. When we tested the RNApred, we found that after a protein sequence was submitted to the server, no prediction results were received from the servers. Therefore, we repeated the same method as Kumar et al.'s work and reconstructed RNApred model based on the Main dataset mentioned in [11]. The reconstructed RNApred was then used to predict RNA-binding proteins in Testset. It correctly predicted 84 out of 144 RNA-binding proteins and 92 out of 144 negatives. The detailed comparison results of the three methods are shown in Table 3. The results demonstrated that our method outperformed those previous methods in the prediction of RNA-binding proteins. The excellent results were due to the effective features and the mRMR-IFS feature selection.

## 4. Conclusions

Accurate identification of new RNA-binding proteins is important to understand RNA-protein interactions. In this study, an accurate method was developed to predict RNA-binding proteins using only sequence information. We proposed three novel features, binding propensity (BP), nonbinding propensity (NBP), and evolutionary information combining with physicochemical properties (EIPP). BP and NBP were constructed based on the prediction results of RNA-binding residues and nonbinding residues, respectively. The EIPP features were improved on those of PSSM by combining evolutionary information with physicochemical properties. The results showed that using those novel features dramatically improved the prediction performance and were effective in distinguishing RNA-binding proteins from nonbinding ones. The mRMR-IFS feature selection method and RF algorithm are then utilized to construct the prediction model. This is the first study in which the mRMR-IFS feature selection method has been successfully used to predict RNA-binding proteins. The prediction model achieved excellent performance, with 86.62% accuracy, 78.34% sensitivity, and 94.91% specificity and an MCC of 0.737. These results indicated that our predictor is a useful tool to predict RNA-binding proteins.

## Figures and Tables

**Table 1:** The prediction performance of the RF model based on various features, evaluated by 10 cycles of 5-fold cross-validation on the MDset dataset.

Feature	Accuracy ± SD	Sensitivity ± SD	Specificity ± SD	MCC ± SD
PSSM-400	0.7967 ± 0.0062	0.7003 ± 0.0093	0.8894 ± 0.0075	0.620 ± 0.016
EIPP	0.8311 ± 0.0105	0.7487 ± 0.0071	0.9107 ± 0.0129	0.662 ± 0.021
CT	0.7482 ± 0.0092	0.6591 ± 0.0067	0.8406 ± 0.0153	0.5096 ± 0.015
EIPP + BP + NBP	0.8428 ± 0.0038	0.7573 ± 0.0082	0.9367 ± 0.0043	0.704 ± 0.008
CT + BP + NBP	0.7661 ± 0.0197	0.7034 ± 0.0132	0.8587 ± 0.0114	0.568 ± 0.026
EIPP + CT	0.8317 ± 0.0139	0.7482 ± 0.0068	0.9202 ± 0.0127	0.671 ± 0.018
EIPP + BP + NBP + CT	0.8573 ± 0.0117	0.7764 ± 0.0143	0.9424 ± 0.0062	0.729 ± 0.020

**Table 2:** Optimal 47 features for prediction of RNA-binding proteins.

Rank	Feature
1	EIPP of ASP in protein sequence for the pKa values of amino group
2	EIPP of GLU in protein sequence for the Balaban index
3	BP(2)
4	EIPP of TYR in protein sequence for the pKa values of amino group
5	CT of class a, class b, and class e
6	CT of class d, class b, and class e

7	EIPP of HIS in protein sequence for the pKa values of amino group
8	EIPP of LYS in protein sequence for the pKa values of carboxyl group
9	CT of class b, class d, and class e
10	CT of class d, class c, and class e
11	EIPP of MET in protein sequence for the molecular mass
12	CT of class b, class e, and class a
13	EIPP of ARG in protein sequence for the pKa values of amino group
14	NBP(2)
15	CT of class c, class e, and class d
16	BP(1)
17	EIPP of TRP in protein sequence for the pKa values of amino group
18	CT of class d, class d, and class e
19	EIPP of LYS in protein sequence for the Balaban index
20	NBP(1)
21	CT of class c, class a, and class d
22	CT of class b, class e, and class d
23	CT of class e, class d, and class e
24	EIPP of HIS in protein sequence for the pKa values of carboxyl group
25	CT of class d, class c, and class f
26	CT of class e, class f, and class d
27	CT of class e, class b, and class d
28	CT of class d, class e, and class c
29	EIPP of GLY in protein sequence for the pKa values of carboxyl group
30	EIPP of THR in protein sequence for the molecular mass
31	CT of class c, class b, and class e
32	CT of class c, class e, and class a
33	EIPP of GLN in protein sequence for Wiener index
34	EIPP of SER in protein sequence for Wiener index
35	EIPP of ASN in protein sequence for the molecular mass
36	CT of class b, class a, and class c
37	CT of class e, class d, and class f
38	CT of class e, class b, and class a
39	EIPP of TRP in protein sequence for the pKa values of carboxyl group
40	CT of class a, class e, and class c
41	EIPP of ARG in protein sequence for the lowest free energy
42	CT of class e, class c, and class d
43	EIPP of LYS in protein sequence for the molecular mass

44	CT of class e, class e, and class d
45	EIPP of TYR in protein sequence for Wiener index
46	CT of class e, class c, and class b
47	CT of class f, class c, and class d

**Table 3:** Comparison of the predicted results by our method and some webservers on the Testset.

Method	ACC (%)	SE (%)	SP (%)	MCC
Our method	0.7674	0.7222	0.8125	0.537
SVMprot	0.5764	0.7639	0.3889	0.165
RNApred	0.6111	0.6389	0.5833	0.223
